# Unveiling a Jejunal Leiomyosarcoma Presenting as a Gastrointestinal Stromal Tumor: A Case Report

**DOI:** 10.7759/cureus.66973

**Published:** 2024-08-15

**Authors:** Mahesh Jadhav, Hariram Nivash T

**Affiliations:** 1 Surgery, Dr. D. Y. Patil Medical College, Hospital and Research Centre, Dr. D. Y. Patil Vidyapeeth (Deemed to be University), Pune, IND

**Keywords:** malignancy, chemotherapy, pleomorphic sarcoma, gastrointestinal stromal tumors (gists), leiomyosarcoma, small bowel tumors

## Abstract

Small bowel malignant tumors, particularly leiomyosarcomas (LMSs), are rare and challenging to diagnose due to their asymptomatic nature in the initial stages. The lack of specific symptoms makes it difficult to differentiate LMSs from other gastrointestinal tumors. This report highlights the clinical presentation, diagnostic challenges, treatment, and prognosis of small bowel LMSs, specifically focusing on a rare case of jejunal LMS presented in a 75-year-old man, initially suspected as a gastrointestinal stromal tumor (GIST). Clinical examination, laboratory investigations, imaging studies, and histopathological analysis were performed to diagnose and treat the patient. The patient underwent surgical excision of the tumor with successful recovery. The histopathological analysis confirmed a high-grade LMS, and the patient was advised on adjuvant chemotherapy for further treatment. This case report emphasizes the importance of considering small bowel LMSs in the differential diagnosis of abdominal pain and highlights the challenges in diagnosing and treating these rare tumors. Early detection and appropriate management can improve the prognosis of patients with small bowel LMSs.

## Introduction

Small bowel malignant tumors are uncommon. They make up less than 5% of all malignancies of the gastrointestinal tract. The five types of malignant small bowel tumors include leiomyosarcomas (LMSs) (1.2%), carcinoids (44.3%), adenocarcinomas (32.6%), lymphomas (14.7%), and gastrointestinal stromal tumors (GISTs) (7.2%). Thus, LMSs of the small bowel are incredibly rare. The most prevalent locations are the duodenum (12.6%), ileum (25.2%), and jejunum (32%). LMSs protrude from the mucosa and serosa after emerging from the submucosa [1]. In the initial stages, small intestinal tumors typically present with no symptoms and might be challenging to see with upper and lower endoscopy, whereas, in the latter stages, they may show signs of obstruction, hemorrhage, and perforation, leading to a poor prognosis [2,3]. The overall incidence of smooth muscle tumors, including leiomyomas and LMSs, is about 1 per 100 GISTs. Since there are only a few small case series and isolated reports, the precise incidence of jejunal LMSs is unknown [4]. Between the muscularis propria and mucosa, smooth muscle cells give rise to LMSs, which have a male-to-female ratio of 1:1 to 2:1 [3].

We report a rare case of LMS of the jejunum, presented as a GIST, in a 75-year-old man with complaints of abdominal pain.

## Case presentation

A 75-year-old man presented with intermittent right-sided abdominal pain for 15 days and a lump in the abdomen for one month, without nausea, vomiting, or melena. No history of comorbidities or addictions was reported. On physical examination, the patient was vitally stable, with a palpable lump of 15 x 10 cm in the right lumbar region extending to the right hypochondrium and epigastrium, firm in consistency, with well-defined margins and an upper border palpable separately from the liver, moving with respiration.

**Figure 1:**
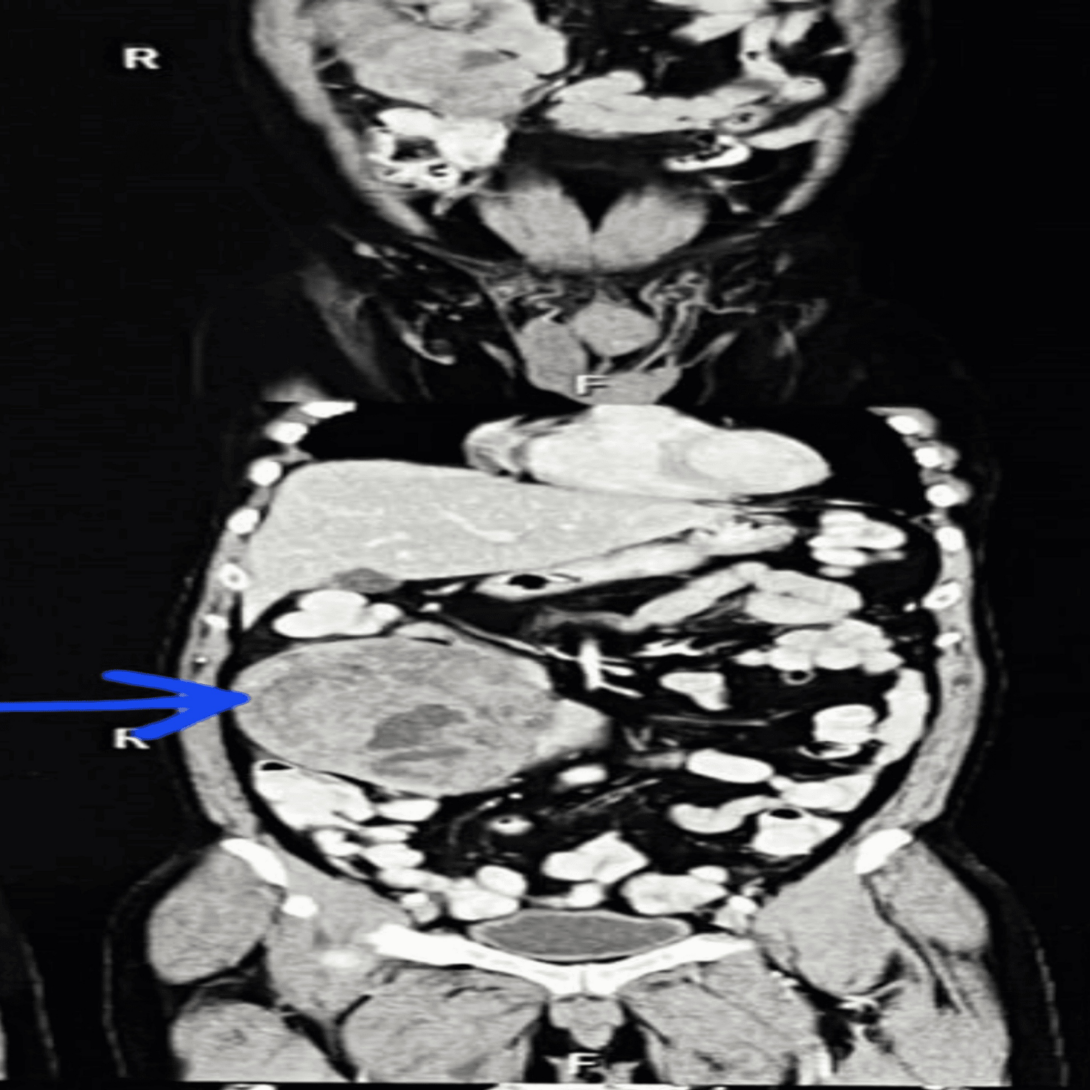
CECT of the abdomen A lobulated, heterogeneously enhancing soft tissue mass, most likely of small bowel origin (arrow). CECT: Contrast-enhanced computed tomography

Laboratory investigations were within the range. Contrast-enhanced computed tomography (CECT) of the abdomen revealed a 13.4 x 11.3 x 9.1 cm well-defined, soft tissue density lesion, showing heterogeneous post-contrast enhancement with multiple areas of hypodensity and a few areas of hyperdensity, suggestive of necrosis and hemorrhage, with focal loss of fat planes involving the jejunal loops (Figure 1).

**Figure 2:**
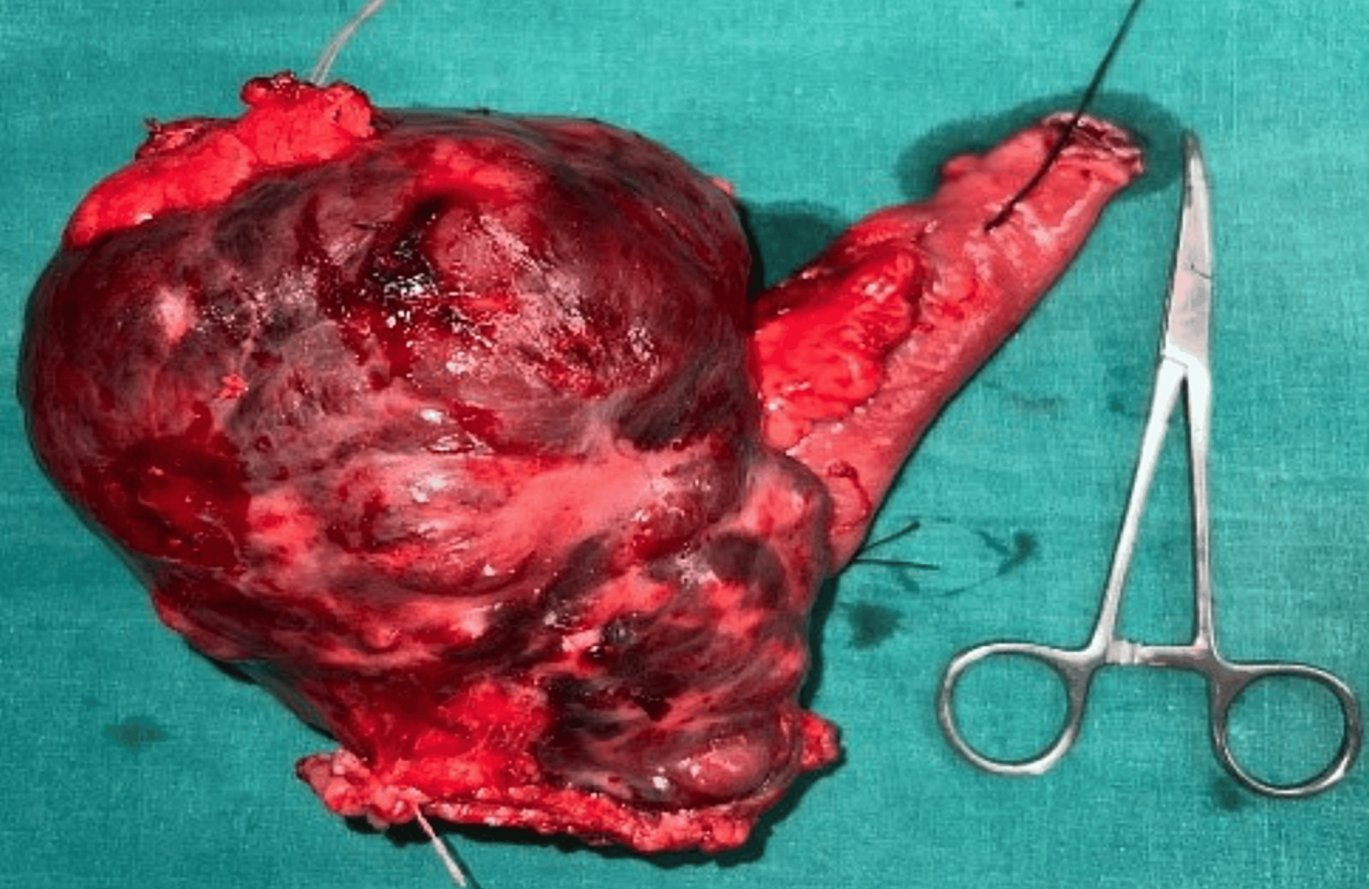
Gross specimen

GIST was clinically suspected. The carcinoembryonic antigen (CEA) level was 1.17 ng/mL. The Tru-cut biopsy was suggestive of pleomorphic sarcoma. Laparotomy revealed a 15 x 10 cm mass arising from the mesenteric border of the jejunum, 60 cm from the duodenojejunal (DJ) flexure, with omental and transverse mesocolic adhesions (Figure 2).

**Figure 3:**
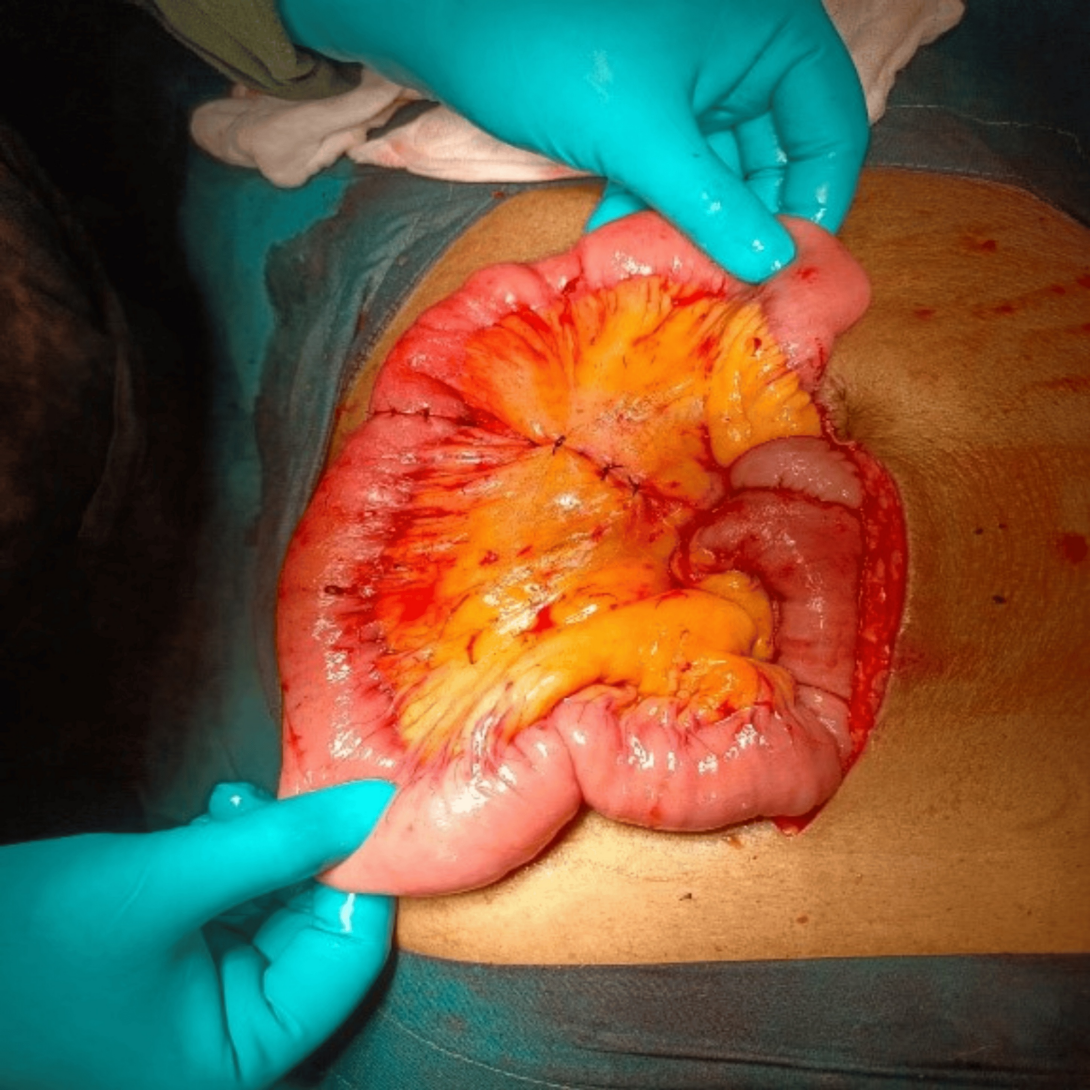
Anastomosis of the jejunum

The liver, omentum, peritoneum, and bowel were normal. Excision of the mass, with resection and anastomosis of the jejunum with a 5 cm margin clearance, was performed (Figure 3).

Gross examination revealed an encapsulated, globular tumor attached to the mesenteric fat, measuring 18 x 10 x 7 cm. Microscopy revealed tumor cells with free jejunal margins. The initial pathology report suggested a malignant spindle cell tumor, which prompted further subtyping via immunohistochemistry (IHC). The tumor cells were positive for smooth muscle actin (SMA) and calponin, whereas they were negative for cluster of differentiation (CD)-34 and CD-117, confirming the diagnosis as high-grade LMS.

The patient made an excellent recovery postoperatively. He was advised to undergo docetaxel and gemcitabine regimens and was instructed to follow up for additional care.

## Discussion

Jejunal LMSs are extremely rare. GISTs make up the majority of all small intestine mesenchymal tumors. The overall incidence of smooth muscle tumors, including leiomyomas and LMSs, is about 1 per 100 GISTs. Since there are only a few small case series and isolated reports, the precise incidence of jejunal LMSs is unknown [4]. Between the muscularis propria and mucosa, smooth muscle cells give rise to LMSs, which have a male-to-female ratio of 1:1 to 2:1 [3]. Usually, they are asymptomatic. Patients may experience fatigue, malaise, melena, and chronic anemia, in addition to chronic abdominal pain [1,3]. The atypical clinical presentation of LMSs makes diagnosis challenging with colonoscopy and esophagogastroduodenoscopy (OGD). Most tumors are found at an advanced stage. The uterus, abdomen, retroperitoneum, and major blood vessels are common anatomical sites. With a preference for the thigh, LMSs of the extremities account for 10-15% of limb sarcomas and are less prevalent [5].

It is still exceedingly difficult to differentiate benign from malignant small intestinal cancers preoperatively, as 40% of these cancers are found by accident. Multiple exams should be combined to diagnose LMSs due to their unusual clinical symptoms. To diagnose intestinal LMSs, CECT, magnetic resonance imaging (MRI)-enterography, and enteroclysis are helpful. A size larger than 6 cm and the presence of uneven borders, peripheral lymphadenopathy included or not, suggest a high probability of malignancy, most likely a GIST or the less common LMS [6]. Because CT imaging is quick, inexpensive, and highly sensitive in identifying the cause of gastrointestinal bleeding, it offers advantages over MRI in acute settings [1]. Furthermore, it is also capable of successfully locating metastases. However, MRI may be more effective than CT at identifying malignancies and small lesions without the need for ionizing radiation [7]. Better soft tissue delineation and increased sensitivity in identifying mucosal lesions are two benefits of MRI, which also offers the ability to distinguish between various malignancies based on T1 and T2 characteristics [1]. The benefit of multidetector CT (MDCT) with non-ionic contrast, or CT enteroclysis/enterography, is that it can identify distant metastasis, the extent of mesenteric involvement, potential transmural extension, and the true extent of wall lesions all in one go. The advantages of MDCT over MRI are quick imaging, low cost, and excellent detection sensitivity. Other techniques, such as enteroscopy and capsule endoscopy, are less accurate at defining mucosal alterations with gut wall or submucosal expansions. Positron emission tomography is not commonly used but can be useful in assessing metastases [8]. The presence of LMSs for preoperative identification was examined in a thorough analysis of several studies by examining the possible relationship between clinical markers, such as D-dimer, C-reactive protein (CRP), cancer antigen-125 (CA-125), and lactate dehydrogenase (LDH). The fact that atypical tumor markers are comparatively uncommon in small bowel LMSs should not be overlooked, though. Moreover, there isn’t any agreement on the part of CA-125 in LMS diagnosis. Nevertheless, higher serum levels of LDH and CA-125 may aid in the exact diagnosis of LMSs when paired with imaging findings [6].

From a histological perspective, the primary antibodies used to identify GISTs are CD-117 (also known as c-KIT), discovered on GIST-1 (DOG1), and CD-34, while the antibodies for LMSs are SMA, desmin, caldesmin, and calponin [8]. Since GISTs and LMSs share a similar morphologic appearance, immunohistochemical techniques should be used to distinguish between these cancers. The positivity of SMA and desmin and the negativity of CD-117, DOG1, and CD-34 set LMSs apart from GISTs [1,3,4]. For soft tissue sarcomas, the tumor can be graded using the French or Trojani methods (FNCLCC). In the context of high-grade malignancies, 10 or more mitoses per 50 high-power fields (HPFs) are seen [8].

Radial surgical excision is still the primary treatment for all small intestinal tumors. Metastases are not a frequent finding given the percentage of individuals with delayed presentations. Since lymph node metastases are rare in LMSs, advanced lymph node dissection is usually not required during these surgeries [6]. Adjuvant chemotherapeutic drugs have not been demonstrated to be beneficial in the treatment of small intestinal illnesses; however, they are used for GISTs and uterine LMSs [2]. Chemotherapy, either with gemcitabine or doxorubicin-based regimens in the first-line instance, is usually administered to patients with advanced LMSs. Doxorubicin with dacarbazine is another combination used in early-line therapy for LMSs. Pazopanib, trabectedin, and other chemotherapy drugs are used in the later stages of treating LMSs. Patients with advanced liposarcomas (LPSs) or LMSs who have previously received anthracycline treatment are eligible for trabectedin [5]. Sadly, adjuvant radiation therapy has not proven effective in treating LMSs due to their widely recognized radioresistance. Patients who have had a full resection are closely monitored, and adjuvant therapy has nothing to do with this process. Because these tumors are physiologically diverse and aggressive, periodic ultrasound (USG) or CT scans are advised [9].

Small intestinal LMSs have an extremely poor prognosis. The five-year survival rate for low-grade disease is 55%, whereas the rate for high-grade disease is 5-20% [3].

## Conclusions

Diagnosing jejunal LMSs, particularly when they present with features suggestive of gastrointestinal tumors, is challenging. The rarity of LMSs, combined with their asymptomatic nature in the early stages and overlapping clinical presentations with other GISTs, complicates accurate diagnosis. Advanced imaging techniques, histopathological evaluation, and immunohistochemical analysis are crucial for differentiation and confirmation. The successful surgical resection of the tumor in this case highlights the importance of prompt and precise surgical intervention, which remains the cornerstone of treatment for small bowel LMSs. Adjuvant chemotherapy, tailored to the individual patient's needs, can further enhance treatment outcomes. Despite the challenges, early detection and comprehensive management can significantly improve prognosis, even for high-grade malignancies. This report emphasizes the need for heightened clinical awareness and a multidisciplinary approach to manage and treat rare tumors like jejunal LMSs effectively, ensuring better patient outcomes through early intervention and personalized care.
